# Antimicrobial Resistance Profile of *Staphylococcus hyicus* Strains Isolated from Brazilian Swine Herds

**DOI:** 10.3390/antibiotics11020205

**Published:** 2022-02-06

**Authors:** Andrea Micke Moreno, Luisa Zanolli Moreno, André Pegoraro Poor, Carlos Emilio Cabrera Matajira, Marina Moreno, Vasco Túlio de Moura Gomes, Givago Faria Ribeiro da Silva, Karine Ludwig Takeuti, David Emilio Barcellos

**Affiliations:** 1 Department of Preventive Veterinary Medicine and Animal Health, Faculty of Veterinary Medicine and Animal Science, University of São Paulo, São Paulo 05508-270, Brazil; luzanolli@gmail.com (L.Z.M.); andrepegoraro21@gmail.com (A.P.P.); marinamo@gmail.com (M.M.); gomesvtm@gmail.com (V.T.d.M.G.); givagofaria@yahoo.com.br (G.F.R.d.S.); 2 Facultad de Ciencias Básicas, Universidad Santiago de Cali, Cali 760042, Colombia; k.rlos89.cabrera@gmail.com; 3 Setor de Suínos, Faculdade de Veterinária, Universidade Federal do Rio Grande do Sul (UFRGS), Porto Alegre 91501-970, Brazil; karinelt87@yahoo.com.br (K.L.T.); davidbarcellos@terra.com.br (D.E.B.)

**Keywords:** *Staphylococcus hyicus*, antimicrobial resistance, PFGE, exudative epidermitis, swine

## Abstract

*Staphylococcus hyicus* is the causative agent of porcine exudative epidermitis. This disorder affects animals in all producing countries and presents a widespread occurrence in Brazil. This study evaluated strains from a historical collection in order to detect the presence of exfoliative-toxin-encoding genes (SHETB, ExhA, ExhB, ExhC, ExhD), characterize the strains using PFGE, and determine their respective antimicrobial resistance profiles. The results obtained from the evaluation of 77 strains from 1982 to 1987 and 103 strains from 2012 reveal a significant change in resistance profiles between the two periods, especially regarding the antimicrobial classes of fluoroquinolones, amphenicols, lincosamides, and pleuromutilins. The levels of multidrug resistance observed in 2012 were significantly higher than those detected in the 1980s. It was not possible to correlate the resistance profiles and presence of genes encoding toxins with the groups obtained via PFGE. Only 10.5% of the strains were negative for exfoliative toxins, and different combinations of toxins genes were identified. The changes observed in the resistance pattern of this bacterial species over the 30-year period analyzed indicate that *S. hyicus* could be a useful indicator in resistance monitoring programs in swine production. In a country with animal protein production such as Brazil, the results of this study reinforce the need to establish consistent monitoring programs of antimicrobial resistance in animals, as already implemented in various countries of the world.

## 1. Introduction

*Staphylococcus hyicus* is the causative agent of exudative epidermitis in pigs, a generalized cutaneous infection characterized by skin exfoliation, excessive sebaceous secretion, and the formation of a brownish coat of exudate that may cover the entire body [1]. The disease is a widely recognized condition in pigs, especially in suckling and weaned piglets. It has been sporadically reported to cause significant morbidity that can be up to 90% in infected herds, and moderate mortality in naïve herds [2].

*S. hyicus* strains may be considered pathogenic or nonpathogenic according to their ability to induce exudative epidermitis in pigs and their ability to produce the exfoliative toxins, which are the main virulence factors necessary to induce the disease [3]. Until now, five exfoliative toxins from *S. hyicus* were described. SHETB was characterized in Japan [4], and ExhA, ExhB, ExhC, and ExhD were characterized in Denmark [5]. The Exh toxins have been shown to cause a loss of cell adhesion in the epidermis of porcine skin by cleaving desmoglein-1, while human desmoglein-1 is resistant to *S. hyicus* exfoliative toxins [3,6,7].

The disease is frequently treated with antimicrobial agents, but treatment is a problem because of the frequent occurrence of antimicrobial resistance in pig strains and subsequent treatment failure. The frequent occurrence of antimicrobial resistance has been previously reported among *S. hyicus* in different countries [1]; in contrast, there is limited information regarding the distribution of different resistance profiles of *S. hyicus* originating in Brazilian swine.

This investigation evaluated *S. hyicus* strains from Brazilian pigs with exudative epidermitis, examined in two different periods with an interval of 30 years, for the purpose of detecting the presence of genes encoding exfoliative toxins, characterizing the strains using PFGE, and determining the minimal inhibitory concentration of antimicrobial agents against each strain.

## 2. Materials and Methods

### 2.1. Bacterial Isolation and Culture Conditions

A total of 77 *S. hyicus* strains isolated in the 1980s and 103 strains isolated in 2012 were evaluated. The strains were isolated from skin lesions of pigs presenting with exudative epidermitis in 27 swine herds from two states, Rio Grande do Sul and São Paulo. Skin swabs were inoculated onto Tween 80 agar plates and aerobically incubated for 18–24 h at 37 °C [8]. Colonies with morphological characteristics of *S. hyicus* were selected and identified using standard biochemical procedures [9] and polymerase chain reaction (PCR). Historical strains were stored in a lyophilized form following isolation, whereas recent strains were stored at −86 °C until characterization.

### 2.2. Detection of Genes Encoding Superoxide Dismutase A and Toxins SHETB, ExhA, ExhB, ExhC, and ExhD

All strains were subjected to species-specific PCR with partial amplification of the *sodA* gene that encodes superoxide dismutase A, as previously described [10], to confirm the identification of *S. hyicus*. Genes encoding toxins SHETB, ExhA, ExhB, ExhC, and ExhD were detected as described in [5,11].

Purified DNA was recovered according to the protocol of Boom et al. [12], with previous enzymatic treatment for 60 min at 37 °C, with 100 mg of lysozyme and 20 mg of proteinase K (USBiological, Swampscott, MA, USA). Samples were stored at −20 °C until processing.

Polymerase chain reactions (50 µL) comprised 5 µL of genomic DNA, ultrapure water, 10× PCR buffer, 1.5 mM MgCl_2_, 200 µM of dNTPs, 10 pmol of each primer, and 1.25 U of Taq-DNA-polymerase (Fermentas Inc., Rockville, MD, USA). The amplified products were stained with BlueGreen^®^ (LGC Biotecnologia, São Paulo, Brazil) and separated by electrophoresis using 1.5% agarose gel, using the 100 bp DNA Ladder^®^ (New England Biolabs Inc., Ipswich, MA, USA).

### 2.3. Molecular Typing by PFGE

*S. hyicus* strains were grown in brain heart infusion broth for 18–24 h at 37 °C. Plug preparation and DNA extraction followed a previously described protocol [13]. The restriction enzyme *SmaI* (New England Biolabs, Ipswich, MA, USA) was used for DNA digestion at 30 °C for 24 h. Electrophoresis was performed using 1% SeaKem Gold^®^ agarose (Cambrex Bio Science Rockland, East Rutherford, NJ, USA) and a CHEF-DR III System (Bio-Rad Laboratories, Hercules, CA, USA) with 0.5× TBE at 14 °C. DNA fragments were separated at 6 V/cm at a 120° fixed angle, with pulse times from 3 to 33 s ramping for 20 h. Gels were stained with a fluorescent DNA stain (SYBR^®^ Safe, Invitrogen Corporation, Carlsbad, CA, USA) for 30 min and imaged under UV transillumination. Lambda DNA-PFGE marker (New England Biolabs, Ipswich, MA, USA) was used for fragment size determination.

### 2.4. Broth Microdilution

The minimal inhibitory concentration (MIC) was determined by the broth microdilution technique as recommended by the Clinical and Laboratory Standards Institute [14], using Sensititre™ Standard Susceptibility MIC Plates BOPO6F (TREK Diagnostic Systems/Thermo Fisher Scientific, Waltham, MA, USA). *Staphylococcus aureus* (ATCC 29213) was used as a quality control. The applied breakpoints for interpretation of results were obtained mainly from the CLSI supplement VET08 [14] and are described in Table 1. If interpretive criteria were not present in the VET08 dataset [14], applied breakpoints were calculated using the twenty-eighth edition of the CLSI performance standard M100 [15], and the literature [16,17]. The reported breakpoints were selected with the following order of preference: those described for swine species were favored, then those for *Staphylococcus* spp. (regardless of the animal species or human indication), and in cases where there was no description in the CLSI or EUCAST datasets, a literature reference was used.

### 2.5. Statistical Analysis

The association analysis between resistance profile and strain origin was performed with SPSS 16.0 (SPSS Inc., Chicago, IL, USA), using chi-square and Fisher’s exact tests. Statistical significance was considered when *p*-values were less than 0.05. The PFGE fingerprint patterns were analyzed by a comprehensive pairwise comparison of restriction fragment sizes, using the Dice coefficient. The mean values obtained from the Dice coefficient were employed in UPGMA, using BioNumerics 7.6 (Applied Maths NV, Sint-Martens-Latem, Belgium). The isolates were considered from different pulsotypes when they differed by four or more bands [18]. Resistance profiles were analyzed as categorical data with the Dice coefficient, using BioNumerics 7.6 software (Applied Maths NV, Sint-Martens-Latem, Belgium).

## 3. Results

All strains from this study were confirmed as *S. hyicus* by PCR. The detection of exfoliative-toxin-encoding genes resulted in the following frequencies: shetB 0%, exhA 34.4% (62/180), exhB 24.4% (44/180), exhC 76.1% (137/180), and exhD 54.4% (98/180). By considering the distribution of toxin genes according to the year of isolation, it was possible to observe a significant increase in the occurrence of the ExhA toxin and a significant reduction in the occurrence of the ExhB toxin between strains from the 1980s and 2012 (Table 2). Only 10.5% (19/180) of strains were negative for all toxin genes, and 13 different profiles were identified according four toxins detected.

**Figure 1:**
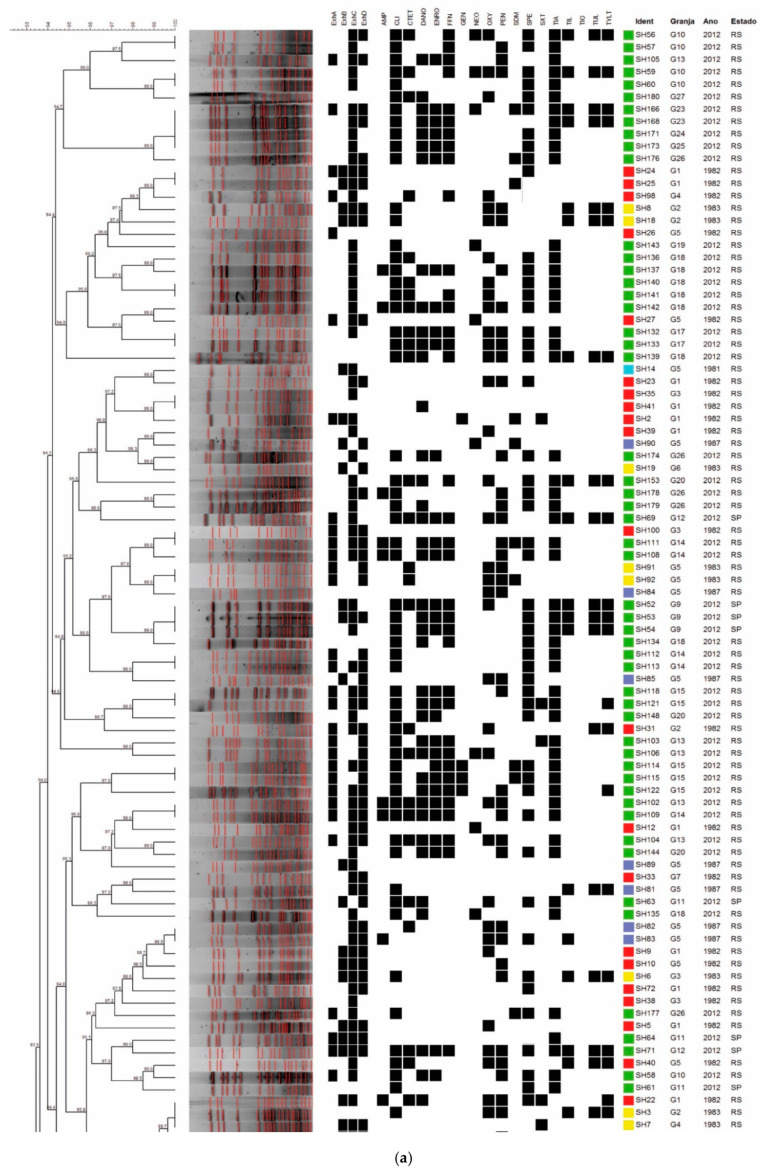

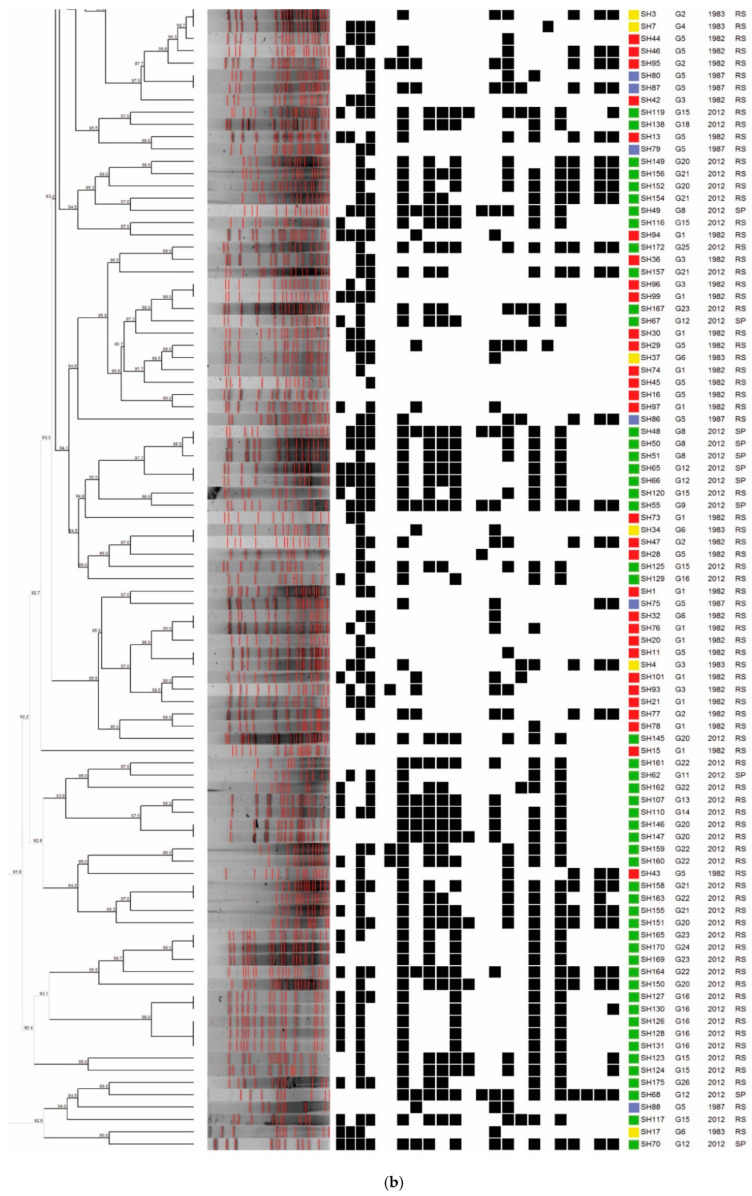
(**a**) Dendrogram showing the relationship among the *S. hyicus* pulsotypes, resistance profiles, and detection of toxin genes (Part I). (**b**) Dendrogram showing the relationship among the *S. hyicus* pulsotypes, resistance profiles, and detection of toxin genes (Part II).

Tests of 180 strains yielded 123 profiles through PFGE with the SmaI enzyme, presenting 9 to 20 fragments with sizes ranging from 40 to 300 kb. Strains showed a similarity greater than 70%, and it was possible to identify 28 pulsotypes. In several cases, pulsotypes grouped strains from the same farm, period of isolation, or state of origin. The dendrogram shown in Figure 1a,b illustrates the results observed in the PFGE analysis.

The observed pulsotypes were denoted as C1 to C28. Several pulsotypes clearly grouped the strains isolated in 2012, such as clusters C1, C2, C18, C19, C20, C23, C25, and C27. Other pulsotypes grouped strains isolated in the 1980s, such as clusters C7, C8, and C21. By considering the farm of origin, it was possible to observe the presence of strains from the same farm clustering into certain groups, such as cluster C17, which contained six strains from farm 18. However, other strains from this farm can also be found in groups C2, C10, and C16. This behavior is repeated in strains from different farms and can be observed most clearly at extreme points of the dendrogram, where strains from farm 15 are allocated into clusters C1, C6, C11, C13, C15, C26, and C27. It was not possible to correlate the resistance profiles, the presence of toxin-encoding genes, and the state of origin with the PFGE clusters.

**Figure 2:**
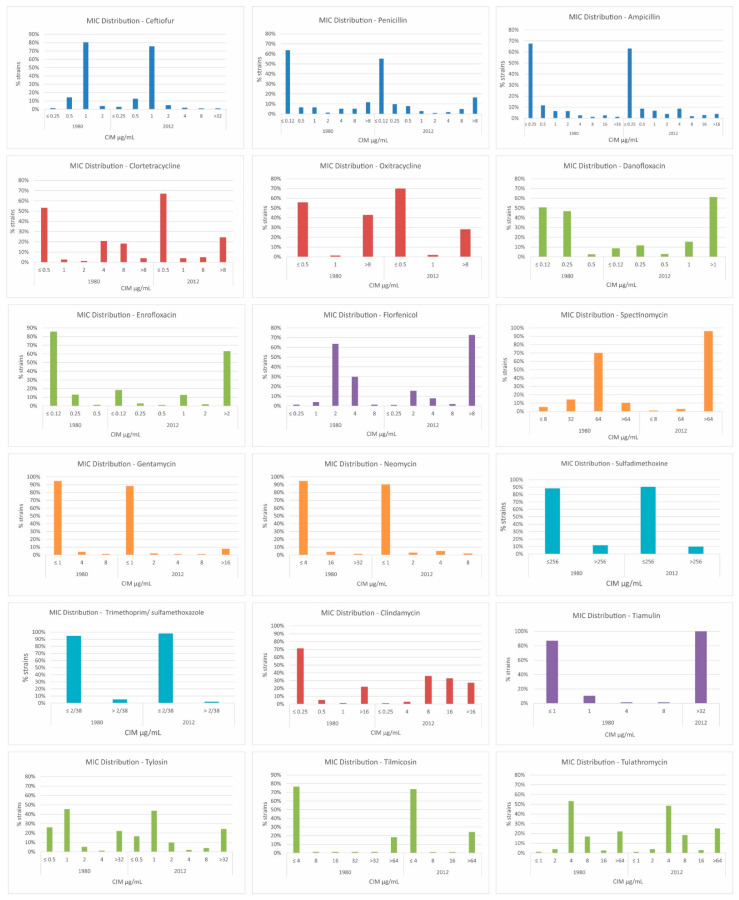
Distribution of MIC values according to antimicrobial tested and period evaluated (1980s or 2012).

All strains were subjected to the determination of the minimum inhibitory concentration; the observed resistance rates are presented in Table 3 and Figure 2. It is possible to observe that the resistance pattern changed when comparing strains from the 1980s to 2012 (a period of 30 years), particularly when considering the antimicrobial classes of fluoroquinolones, lincosamides, pleuromutilins, and amphenicols. According to the resistance phenotype, strains were classified into 86 resistance profiles, with 103 strains from 2012 classified into 55 profiles, and 77 strains from 1982 to 1987 into 31 profiles.

Multidrug-resistant strains (resistant to three or more different antimicrobial classes) were found in both assessed groups. However, we observed that the frequency of multidrug-resistant strains isolated in 2012 was significantly higher (*p* < 0.001) than that of those isolated between 1982 and 1987 (Table 4). Among multidrug-resistant strains, there was wide variation in the number of antimicrobial classes against which the strains presented resistance between the studied periods. Among the strains from 1982 to 1987, there were no strains which were resistant to more than six antimicrobial classes, whereas in the 2012 group, 25% of tested strains were resistant to more than seven antimicrobial classes. The distribution of MIC values (MIC50 and MIC90) from the historic and 2012 strains is presented in the Supplementary Material (Tables S1 and S2).

## 4. Discussion

Exudative epidermitis has been described in swine for over 170 years, and its impact on pig production is observed to this day in different countries worldwide. The analysis of genes encoding exfoliative toxins presented here revealed a high frequency of positive samples for one or more toxins. Few studies have described the frequency of *S. hyicus* toxigenic strains. In a study carried out in Japan, Futagawa-Saito et al. [19] described the following rates: ExhA 35.7% (74/207), ExhB 19.3% (40/207), ExhC 0.5% (1/207), and ExhD 16.9% (35/207). Their results are similar to those observed in this study, except for the ExhC toxin, whose gene was detected more frequently in Brazilian strains (76.1%). Andresen [20] described the detection of genes in 218 *S. hyicus* strains from different countries (across Europe, Japan, and EUA), observing the following frequencies: ExhA 10.6% (23/218), ExhB 5.5% (12/218), ExhC 3.2% (7/218), and ExhD 14.2% (31/218). The increase in the frequency of the ExhA toxin and the reduction in the ExhB toxin observed between the periods evaluated in this study have no similar descriptions in the literature.

Through PFGE, the evaluated strains showed a high genetic diversity, which is characteristic of well-adapted agents which are widely disseminated in the population. *S. hyicus* strains constitute part of the microbiota of healthy animals, even if these strains have been isolated from animals with a clinical picture of epidermitis. Few studies report the application of PFGE in the characterization of *S. hyicus* strains. The most striking association in the different clusters formed in this study is related to the period of isolation. Some pulsotypes stand out for grouping strains isolated in 2012, such as clusters C1 (six strains), C2 (six strains), C18 (five strains), C19 (six strains), C20 (four strains), C22 (six strains), C23 (six strains), C25 (eight strains), and C27 (six strains). That is, 53 of 103 strains from 2012 (51.4%) were clustered according to the isolation period. Other pulsotypes contained only those strains isolated in the 1980s, such as clusters C7 (8 strains), C8 (9 strains), and C21 (4 strains), totaling 21 of 77 strains from the 1980s (27.2%), grouped in common clusters.

It was also possible to observe some groups such as C3, C4, C12, and C15 in which there was a predominance of strains from the 1980s, but with the presence of some recent strains. The strains from certain farms tend to be grouped into specific pulsotypes; however, in all cases, specific isolates from each farm are dispersed at different points on the dendrogram. It was not possible to correlate the resistance profiles and presence of genes encoding toxins with the groups obtained in PFGE.

The MICs of the 180 studied strains were evaluated against 9 antimicrobial classes, and the rising resistance level in several of these classes certainly reflects the increase in antibiotic use in intensive pig production systems in Brazil in recent decades [21]. This indicates that *S. hyicus* could be an important bacterial species for use in antimicrobial resistance monitoring programs in pig production in Brazil, as has been achieved in Denmark [1].

Among the tested beta-lactams, we saw a slight increase in ampicillin and penicillin resistance rates over the 30 years evaluated. The frequency of ceftiofur resistance was low in both groups. This may be related to the high cost of ceftiofur what restricted its use via parenteral administration until 2012. Nevertheless, higher rates of ceftiofur resistance have been described in Canada, where Park et al. [22] described 71% (101/142) of assessed *S. hyicus* strains as ceftiofur resistant in a study conducted on 30 herds. The occurrence of *S. hyicus* resistance to penicillin and ampicillin, or to ampicillin, penicillin, and ceftiofur, and positivity for the presence of the *mecA* gene was also described in the same study and has been considered a risk for swine and human health [22].

A slight decrease in tetracycline resistance rates was also observed in the studied strains. The culmination of the use of tetracyclines in Brazilian swine production was in the 1980s and 1990s. However, a study conducted in 2017 [21] showed that this class was the third most used among 25 Brazilian swine herds, despite the widespread resistance genes among several bacterial species.

The tetracycline resistance rates are quite varied in the literature, according to different countries and studied periods. In Denmark, Wegener et al. [23] reported that 44% (44/100) of *S. hyicus* strains were tetracycline resistant between 1991 and 1992, while Aarestrup and Jensen [1] reported that only 28.9% (109/377) of Danish *S. hyicus* strains were tetracycline resistant in 2002. In Canada, 55% (79/142) of *S. hyicus* strains were resistant to tetracycline [22], whereas only 1.4% (3/207) of strains were resistant to doxycycline in a Japanese study [19].

The fluoroquinolone resistance levels found were low but represented a significant increase among the studied strains. This change probably reflects the introduction of these antimicrobial in swine production in the 1990s and the selection of resistant strains since then. Our results corroborate previous reports of low levels of enrofloxacin (5.6%) and ciprofloxacin (4.8%) resistance described in Danish *S. hyicus* [1].

The tested aminoglycosides exhibited two distinct resistance patterns: gentamicin and neomycin had low levels of resistance in the 1980s, and these rates have not increased significantly since then, but spectinomycin presented a low resistance rate in the 1980s which has significantly increased as of 2012. Spectinomycin has been added to in-feed formulations for several years to aid the control of enteric infections; this does not occur with neomycin and gentamicin, which are mostly restricted to individual treatments in oral or injectable formulations in Brazil. High resistance rates against spectinomycin (45.1%) have also been described in Canada [22].

The amphenicols currently permitted for use in swine production in Brazil are florfenicol and thiamphenicol. The use of florfenicol in the country began in the 2000s and has intensified since then, especially in in-feed treatment formulations. Resistance levels of the strains isolated in the 1980s were extremely low but demonstrated a large increase in 2012. In a Danish study conducted in 2003, resistance rates to florfenicol were 0% [24]. The sulfonamides, represented by sulfadimethoxine and a combination of trimethoprim/sulfamethoxazole, presented low levels of resistance in strains from the 1980s compared to a slight reduction in 2012. The drop in resistance is probably due to the reduction in the use of sulfonamides in feed production animals in Brazil during some years.

The studied macrolides (tilmicosin, tylosin, and tulathromycin) presented only a slight increase in resistance levels, despite their wide use in the treatment of respiratory and enteric infections in intensive swine production. In the literature, among the macrolides, erythromycin resistance rates were most frequently described with a variation of 15% to 62% between 1991 and 2001, in *S. hyicus* isolated in Denmark [1,23].

For clindamycin, we found high resistance rates in both studied periods. Regarding lincosamide resistance, a 59% (59/100) lincomycin resistance rate was reported in Denmark in 1990 [25]. The combination of lincomycin and spectinomycin has been widely used in recent years to control and prevent respiratory and enteric infections in Brazilian swine production. This could explain the high resistance rates to both of these antimicrobial classes during the studied periods.

The pleuromutilin class, represented by tiamulin, was approved for use in pigs in 1979 in Europe and the United States and was introduced in Brazil in late 1990. The drug is the second most used during the weaning and growing phases, as described by Dutra et al. [21], reinforcing the observed result that tiamulin resistance rates have increased from 0% in the 1980s to 100% of the strains in 2012. In staphylococci, the transferable resistance mechanisms of pleuromutilins have been linked to vga genes, which codify the ABC transporter that exports pleuromutilins, streptogramin A, and lincosamides. There are seven vga resistance genes described thus far (vga A, B, C, D, and E) and all of them are located in plasmids [26]. It is suggested that the use of pleuromutilins may have also favored the selection of cfr-positive *Staphylococcus* of animal origin, and a high frequency of multidrug resistance in strains resistant to pleuromutilins has been observed. Mobile elements containing pleuromutilin resistance genes often contain genes encoding resistance to other antimicrobial classes. Not only does the use of pleuromutilins select strains with this set of resistance genes, but also the use of other antimicrobial classes can select pleuromutilin-resistant strains in a mutual selection process [27]. Considering these risks of cross-selection, in January 2020, the Brazilian government prohibited the use of lincomycin, tiamulin, and tylosin as growth promoters in animals [28].

Given the genetic components related to resistance against the multiple antimicrobial classes tested in this study, and the associations described in the literature among different genes and mobile elements, it becomes easier to understand the observed changes in the resistance profiles of *S. hyicus* strains over the 30-year period studied and the significant increase in the phenomenon of multidrug resistance.

## 5. Conclusions

The results described here expand current knowledge about porcine exudative epidermitis in Brazil, as well as painting a portrait of the change in the antimicrobial resistance profiles that can occur over time in a bacterial population. The selection of multidrug-resistant *S. hyicus* strains in swine in this 30-year interval suggests that this phenomenon may also be occurring in other Gram-positive bacterial species of greater zoonotic potential such as *S. aureus, Enterococcus faecalis*, or *E. faecium*.

## Figures and Tables

**Table 1:** Antimicrobials’ MIC range evaluated, and breakpoints applied to *S. hyicus*.

Antimicrobial	MIC Range (µg/mL)	MIC Breakpoints		
		Susceptible	Intermediary	Resistant
Ampicillin	≤0.25–1.0	≤0.25	0.5	≥1
Ceftiofur	≤0.25–2.0	≤2	4	≥8
Penicillin	≤0.12–2.0	≤0.12	-	≥0.25
Chlortetracycline	≤0.5–>8.0	≤0.5	1	≥2
Oxitetracycline	≤0.5–>8.0	≤0.5	1	≥2
Danofloxacin	0.5–>1.0	≤0.25	0.5	≥1
Enrofloxacin	0.5–>2.0	≤0.5	1	≥2
Florfenicol	1.0–>8.0	≤2	4	≥8
Spectinomycin	16.0–>64.0	≤32	64	≥128
Gentamycin	≤1.0–>16.0	≤2	4	≥8
Neomycin	≤4.0–>32.0	≤8	-	-
Sulfadimethoxine	>256.0	≤256	-	≥512
Trimethoprim/sulfamethoxazole	>2/38	≤2/38	-	≥4/76
Clindamycin	≤0.25–>16.0	≤0.5	1–2	≥4
Tylosin	≤0.5–>32.0	≤1	2–4	˃4
Tilmicosin	≤4.0–>64.0	≤16	-	≥32
Tulathromycin	≤1.0–>64.0	≤16	32	≥64
Tiamulin	1.0–>32.0	≤16	-	≥32

**Table 2:** Frequency of strains positive for exfoliative toxins in the two periods evaluated (1980s and 2012).

Toxins	1980		2012		*p*
	N	%	N	%	
ExhA	17	22.08	45	43.68	<0.001
ExhB	31	40.26	13	12.62	<0.001
ExhC	55	71.40	82	79.61	0.150
ExhD	48	62.30	50	48.54	0.087

*p*—probability of the chi-square test or Fisher’s exact test (£).

**Table 3:** Resistance rates of *S. hyicus* from the 1980s and 2012 against tested antimicrobials.

Class	Antimicrobial	1980		2012		*p*
		N	(%)	N	(%)	
Beta-lactams	Ampicillin	16	20.77	29	28.15	0.258
	Ceftiofur	0	0.00	1	0.97	0.386
	Penicillin	28	36.36	46	44.66	0.263
Tetracycline	Oxitetracycline	33	42.86	29	28.16	0.040
	Chlortetracycline	34	44.15	30	29.12	0.037
Fluoroquinolones	Danofloxacin	2	2.60	81	78.64	<0.001
	Enrofloxacin	0	0.00	67	65.05	<0.001
Aminoglycosides	Gentamycin	1	1.30	9	8.74	0.045
	Neomycin	4	5.19	10	9.71	0.263
	Spectinomycin	8	10.4	99	96.1	<0.001
Fenicois	Florfenicol	1	1.30	77	74.76	<0.001
Sulfas	Sulfadimethoxine	9	11.69	10	9.71	0.669
	Trimethoprim/sulfamethoxazole	4	5.19	2	1.94	0.229
Lincosamides	Clindamycin	17	22.08	102	99.03	<0.001
Pleuromutilins	Tiamulin	0	0.00	103	100.00	<0.001
Macrolides	Tilmicosin	16	20.78	25	24.27	0.580
	Tylosin	18	23.38	31	30.10	0.316
	Tulathromycin	17	22.08	26	25.24	0.622

*p*—probability of chi-square or Fisher’s exact (£) tests.

**Table 4:** Frequency of *S. hyicus* strains presenting multidrug resistance to antimicrobials according to isolation period.

Classification	1980		2012		*p*
	N	%	N	%	
Resistant to 2 classes or less	47	61.0	1	1.0	<0.001
Multidrug resistant (3 classes or more)	30	39.0	102	99.0	

*p*—probability of the chi-square test.

## Supplementary Materials

The following supporting information can be downloaded at https://www.mdpi.com/article/10.3390/antibiotics11020205/s1, Table S1: Distribution of MIC values observed in *S. hyicus* strains isolated in 1980 decade. Table S2: Distribution of MIC values observed in *S. hyicus* strains isolated in 2012.
