# Supplementary material for: Antimicrobial Resistance Profile of Staphylococcus hyicus Strains Isolated from Brazilian Swine Herds
Source: Antibiotics (Basel). 2022 Feb 6;11(2):205. doi: 10.3390/antibiotics11020205 (PMC8868439; doi:10.3390/antibiotics11020205)
Supplement: Supplementary file 1 [file antibiotics-11-00205-s001.zip › Table S1a.pdf]

Table S1. Distribution of MIC values observed in *S. hyicus* strains isolated in 1980 decade.

Antimicrobials	Number of strains –1980 decade											MIC*50	MIC*90	Res
MIC(µg/ml)	≤0.12	0.25	0.5	1	2	4	8	16	32	64	>64	(µg/mL)	(µg/mL)	%
Ampicillin	0	52	9	5	5	2	1	2	1	0	0	≤0.25	2	20,7
Ceftiofur	0	1	11	62	3	0	0	0	0	0	0	1	1	0
Clindamycin	0	55	4	1	0	0	0	0	17	0	0	≤0.25	>16	22
Chlortetracycline	0	0	41	2	1	16	14	3	0	0	0	≤0.5	8	44,1
Danofloxacin	39	36	2	0	0	0	0	0	0	0	0	≤0.12	0,25	2,6
Enrofloxacin	66	10	1	0	0	0	0	0	0	0	0	≤0.12	0,25	0
Spectinomycin	0	0	0	0	0	0	4	0	11	54	8	64	>64	10,4
Florfenicol	0	1	0	3	49	23	1	0	0	0	0	2	4	1,3
Gentamicin	0	0	0	73	0	3	1	0	0	0	0	≤1	≤1	1,3
Neomycin	0	0	0	0	0	73	0	3	0	1	0	≤4	≤4	5,2
Oxytetracycline	0	0	43	1	0	0	0	33	0	0	0	≤0.5	>8	42,8
Penicillin	49	0	5	5	1	4	4	9	0	0	0	≤0.12	>8	36,4
Tiamulin	0	0	67	8	0	1	1	0	0	0	0	≤0.5	1	0
Tilmicosin	0	0	0	0	0	59	1	1	1	0	15	≤4	>64	20,7
Tylosin	0	0	20	35	4	1	0	0	0	17	0	1	>32	23,3
Tulathromycin	0	0	0	1	3	41	13	2	0	0	17	4	>64	22

MIC (µg/ml)	≤256	>256	MIC*50 (µg/mL)	MIC*90 (µg/mL)	Res %
Sulfadimethoxine	68	9	≤256	≤256	9.7
**MIC (µg/ml)**	**≤2/38**	**>2/38**			
Cotrimoxazole	73	4	≤2/38	≤2/38	5.2
