# Supplementary material for: Antimicrobial Resistance Profile of Staphylococcus hyicus Strains Isolated from Brazilian Swine Herds
Source: Antibiotics (Basel). 2022 Feb 6;11(2):205. doi: 10.3390/antibiotics11020205 (PMC8868439; doi:10.3390/antibiotics11020205)
Supplement: Supplementary file 1 [file antibiotics-11-00205-s001.zip › Table S2a.pdf]

TableS2. Distribution of MIC values observed in *S. hyicus* strains isolated in 2012.

Antimicrobials	Number of strains - 2012											MIC*50	MIC*90	Res
MIC(μg/ml)	≤0.12	0.25	0.5	1	2	4	8	16	32	64	>64	(μg/mL)	(μg/mL)	%
Ampicillin	0	65	9	7	4	9	2	3	4	0	0	≤0.25	4	28,1
Ceftiofur	0	3	13	78	5	2	0	1	0	0	0	1	1	0,9
Clindamycin	0	1	0	0	0	3	37	34	28	0	0	16	>16	99
Chlortetracycline	0	0	72	2	0	0	0	29	0	0	0	≤0.5	>8	29,1
Danofloxacin	9	12	3	16	63	0	0	0	0	0	0	>1	>1	78,6
Enrofloxacin	19	3	1	13	2	65	0	0	0	0	0	>2	>2	65
Spectinomycin	0	0	0	0	0	0	1	0	0	3	99	>64	>64	96,1
Florfenicol	0	1	0	0	16	8	2	75	0	0	0	>8	>8	74,7
Gentamicin	0	0	0	91	2	1	1	0	8	0	0	≤1	≤1	8,7
Neomycin	0	0	0	0	0	93	3	5	2	0	0	≤4	≤4	9,7
Oxytetracycline	0	0	72	2	0	0	0	29	0	0	0	≤0.5	>8	28,1
Penicillin	57	10	8	3	1	2	5	17	0	0	0	≤0.12	>8	44,6
Tiamulin	0	0	0	0	0	0	0	0	0	103	0	>32	>32	100
Tilmicosin	0	0	0	0	0	76	1	1	0	0	25	≤4	>64	24,3
Tylosin	0	0	17	45	10	2	4	0	0	25	0	1	>32	30
Tulathromycin	0	0	0	1	4	50	19	3	0	0	26	4	>64	25,2

MIC (μg/ml)	≤256	>256	MIC*50 (μg/mL)	MIC*90 (μg/mL)	Res %
Sulfadimethoxine	93	10	≤256	>256	11.7
**MIC (μg/ml)**	**≤2/38**	**>2/38**			
Cotrimoxazole	101	2	≤2/38	≤2/38	1.9
